# Inhaled corticosteroids inhibit substance P receptor expression in asthmatic rat airway smooth muscle cells

**DOI:** 10.1186/1471-2466-12-79

**Published:** 2012-12-17

**Authors:** Miao Li, Yun-xiao Shang

**Affiliations:** 1 Department of pediatrics, Shengjing hospital of China Medical University, Shenyang, 110004, China

## Abstract

**Background:**

Neurokinins (NKs) participate in asthmatic airway inflammation, but the effects of NKs on airway smooth muscle cells (ASMCs) and those of corticosteroids on NKs are unknown.

**Methods:**

To investigate the effect of budesonide on substance P (NK-1) receptor (NK-1R) expression in the lung and ASMCs, 45 Wistar rats were randomly divided into three groups: control, asthmatic, and budesonide treatment. Aerosolized ovalbumin was used to generate the asthmatic rat model, and budesonide was administered after ovalbumin inhalation. On day 21, bronchial responsiveness tests, bronchoalveolar lavage, and cell counting were conducted. NK-1R protein expression in the lung was investigated by immunohistochemistry and image analysis. Primary rat ASMC cultures were established, and purified ASMCs of the fourth passage were collected for mRNA and protein studies via real-time RT-PCR, immunocytochemistry, and image analysis.

**Results:**

NK-1R mRNA and protein expression in the budesonide treatment group rat’s lung and ASMCs were less than that in the asthmatic group but greater than that in the control group.

**Conclusions:**

NK-1R is involved in the pathogenesis of asthma and that budesonide may downregulate the expression of NK-1R in the ASMCs and airways of asthmatic rats, which may alleviate neurogenic airway inflammation.

## Background

Asthma is a chronic inflammatory disease characterized by airway hyper-responsiveness that involves many inflammatory cells and mediators [1]. Neurokinins (NKs) are peptides synthesized by neural tissues that have been implicated as the mediators of neurogenic inflammation in asthma. NKs have potent effects on airway smooth muscle tone, airway secretion, bronchial circulation, and inflammatory and immune cells via the activation of the neurokinin-1 (NK-1R) and neurokinin-2 receptors (NK-2R); as such, they have been proposed to play an important role in human respiratory conditions such as bronchial asthma and chronic obstructive diseases [2]. For example, Pattersson *et al* demonstrated that tachykinin levels were increased in induced sputum from patients with asthma, cough, and acid reflux [3]. In addition, Bai *et al* also demonstrated that tachykinin, NK-1R, and NK-2R mRNA expression is elevated within the airways of asthma patients [4]. Inhaled corticosteroid treatment is the cornerstone of pharmacotherapy for persistent asthma [5], and airway smooth muscle cells (ASMCs) are important in the pathogenesis of this disease; NK-1R and NK-2R expression in human and rat ASMC lung tissue has been confirmed by immunohistochemistry [6,7]. However, the relationship between inhaled corticosteroids and NK-1R expression is unknown, and thus, in our study, we investigated NK-1R expression in asthmatic rat ASMCs to determine the effect of budesonide treatment on neuropeptide receptor expression.

## Methods

### Asthmatic rat model

Forty-five healthy female Wistar rats weighing 150–160 g were purchased from the experimental animal center of China Medical University and divided randomly into three groups: control, asthmatic, and budesonide treatment. All experimental protocols involving animals were approved by the China Medical University Animal Care Committee and complied with the guidelines of the China Council on Animal Care. The modified ovalbumin (OVA) (Sigma-Aldrich, Beijing, China) inhalation method was used to generate the asthmatic rat model as described in detail elsewhere [8]. Briefly, the protocol consisted of a subcutaneous injection of 1 mg of OVA and 200 mg/mL aluminum hydroxide (Sigma-Aldrich, Beijing, China) in 1 mL of PBS and an intraperitoneal injection of 1 mL of heat-killed *Bordetella pertussis* (6 × 10^9^/mL, Beijing, China) on day 0 and day 7. Rats in the control group were treated with 1 mL of PBS containing only 200 mg/mL aluminum hydroxide. Two weeks later, the rats were placed in a transparent glass chamber (approximately 20 cm × 20 cm × 20 cm in volume) connected to an ultrasonic nebulizer (model 100, Yadu, Shanghai, China) and subjected to repeated bronchial allergen challenge via OVA (2%) inhalation for 20 min/day for 6 days. Rats in the control group were challenged with PBS. After OVA inhalation, rats in the budesonide treatment group were given 1 mg of budesonide via inhalation by INQUA NEB plus (PARI) over the course of 5 minutes for 6 days.

### Bronchial responsiveness to methacholine

To investigate OVA-induced effects on airway responsiveness, we measured changes in respiratory parameters in response to methacholine (MCh). After the rats were challenged, they were anesthetized with pentobarbital (30 mg/kg, i.p.), and the trachea was cannulated with a 14-gauge tube. The rats were quasi-sinusoidally ventilated with a computer-controlled small-animal ventilator (flexiVent; SCIREQ, Montreal, Quebec, Canada) with a tidal volume of 8 mL/kg, set automatically depending on body weight at 90 breaths/min and positive end-expiratory pressure of 3.0 cmH_2_O. Airway resistance was measured by the forced oscillation technique. Five doses of MCh (Sigma-Aldrich, Beijing, China) solution (10–160 μg/mL) in 0.5 mL of PBS were given intermittently via jugular vein injection, each 1 min apart. After each MCh challenge, the respiratory system resistance was recorded by animal pulmonary function analysis software, testing baseline airway resistance and Re, which represents changes in airway responsiveness. When Re reached or exceeded the baseline Re 2 times stop to push MCh.

### Bronchoalveolar lavage (BAL) and cell counting

After the lung responsiveness measurements, the rats were disconnected from the ventilator and sacrificed via pentobarbital overdose. A catheter was then inserted into the trachea, and BAL was performed. The cell suspension was concentrated by centrifugation (1000 rpm, 10 min at 4°C), and the cell pellet was resuspended in 1 mL of saline. To perform the differential leukocyte cell count, 0.1 mL of the cell suspension was transferred to a glass slide and stained with Wright-Giemsa stain. A microscope was then used to examine 400 nucleated cells.

### Immunohistochemistry and image analysis

NK-1R protein expression in the lung was detected by immunohistochemistry. Right middle lobes of the lungs were harvested 24 h after the final OVA challenge and fixed in 4% paraformaldehyde, then imbedded in paraffin. Lung sections of 5 μm were cut and blocked with peroxide and non-immune animal serum and incubated sequentially with primary antibody, biotin-labeled secondary antibody, and streptomycin anti-biotin peroxidase. Finally, the sections were stained with DAB, counterstained with hematoxylin, dehydrated, cleared in xylene, and fixed. Negative staining controls were generated by replacing the primary antibody with PBS. The mean density values of NK-1R protein, indicated by brown staining, were calculated in three selected fields under high magnification (×400) via image analysis. Optical density represented the NK-1R protein content in ASMCs.

### ASMC culture

Twenty-four hours after the final challenge, the rats were sacrificed, and primary ASMCs were cultured according to a previously described method [9]. Tracheas were dissected, excised, and washed aseptically. The internal and external membranes of the trachea were removed. The smooth muscles were separated longitudinally from the cartilage and digested in 0.1% trypsin, 0.02% EDTA, and 0.2% type IV collagenase for 30 min in a shaking water bath at 37°C. The harvested cells were collected and cultured in DMEM–F-12 medium (1:1 vol/vol; Thermo Scientific HyClone, Beijing, China) supplemented with 10% FBS (Thermo Scientific HyClone, Beijing, China). The medium was changed every 3 – 4 days. When the ASMCs reached confluency, exhibited an elongated spindle shape, and grew with the typical hill-and-valley appearance, the cells were passaged with a 0.25% trypsin – 0.02% EDTA solution. From then on, passaging was performed every 10 – 14 days, and ASMCs of the fourth passage were used for experiments. ASMCs were identified using anti α-actin (1:200 diluted in PBS; Boster Biotechnology, Wuhan, China) and FITC-conjugated goat-anti-rabbit (1:100, Invitrogen, Beijing, China) and observed under a fluorescence microscope.

### Immunocytochemistry and image analysis

The ASMCs from the different groups were cultured on coverslips, and NK-1R protein expression was detected by immuncytochemistry. After the cells reached confluency, they were fixed in 4% polyphosphate formaldehyde, blocked with peroxide and non-immune animal serum, and incubated sequentially with primary antibody, biotin-labeled secondary antibody, and streptomycin anti-biotin peroxidase. Finally, the ASMCs were stained with DAB, counterstained with hematoxylin, dehydrated, cleared in xylene, and fixed. Negative controls were generated by replacing the primary antibody with PBS. To evaluate positive results, five fields with condensed NK-1R protein expression were selected under low magnification (×100), cell numbers were counted under high magnification (×400), and the mean density values of ASMCs with brown staining were calculated in the five selected fields via image analysis. Optical density represented the NK-1R protein content in ASMCs.

### Real-time RT-PCR analysis

To investigate NK-1R mRNA expression in ASMCs from the various groups, real-time RT-PCR was performed as a quantitative analysis. Total RNA was extracted from ASMC cultures using RNAiso™ Plus reagent (Takara, Dalian, China) and quantified using a spectrophotometer. Following quantification, 2 μg of RNA was reverse-transcribed into cDNA, and real-time quantitative PCR assays were conducted using an ABI PRISM 7500 real-time PCR System (Applied Biosystems, Foster City, CA, USA) and the SYBR PrimeScript™ RT-PCR kit reagent (Takara, Dalian, China). The PCR conditions for NK-1R were 45 cycles of denaturation at 95°C for 5 s and annealing and extension at 60°C for 30 s. Target mRNA levels were normalized to those of GAPDH. The following oligonucleotide primers were used: NK-1R forward 5^′^-CGCCGATGTTTCAGTCCATTC-3^′^, reverse 5^′^-GACGTATTCAGTCCGTGTTGGTTG-3^′^; GAPDH forward 5^′^-GCACCGTCAAGGCTGAGAAC-3^′^, reverse 5^′^-ATGGTGGTGAAGACGCCAGT-3^′^. Gene expression was determined by the 2^−ΔΔCT^ method.

### Statistical analysis

All experiments were repeated in triplicate. All data were expressed as the mean ± SE and analyzed using SPSS 17. One-way analysis of variance with the SNK test was used to test for significance, which was accepted at *P* < 0.05.

## Results

### Airway responsiveness to MCh

**Figure 1:**
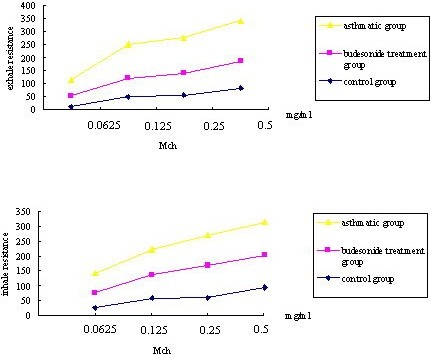
**Airway responsiveness to MCh. **To test the airway responsiveness of asthmatic rats *in vivo*, we measured respiratory parameters induced by MCh. Airway responsiveness of rats in the asthmatic group increased in comparison to the control group after induction by MCh.

To test the airway responsiveness of asthmatic rats *in vivo*, we measured respiratory parameter changes induced by MCh. Airway responsiveness of rats in the asthmatic group increased after induction by MCh when compared to the control group. However, the airway responsiveness of rats in the budesonide treatment group decreased after induction by MCh when compared with the asthmatic group (Figure 1). 

### Inflammatory cells in BAL fluid

The number of inflammatory cells in BAL fluid was measured and compared between the three groups. Remarkably, the total cell number and eosinophils in BAL fluid recovered from OVA-sensitized/challenged rats was significantly higher than that from PBS-treated rats (*P* < 0.05). Conversely, total cells and eosinophils in the budesonide treatment group were significantly decreased when compared with the asthmatic group (*P* < 0.05), but did not significantly differ from the control group (*P >* 0.05) (Table 1). 

**Table 1:** **Inflammation cells in different group rat’s BALF (**Mean **± s) ×10**^**4 **^**/ml**

**Group**	**Total**	**Eosinocyto**	**Lymphocyto**	**Granulocyto**	**Macrophage**
Asthmatic group	610±32^*^	461±31^*^	40±16^*^	20±6.3^*^	88±15^*^
Budesonide treatment group	372±13^#▴^	147±23^#▴^	19±3.5^#▴^	18±3^#▴^	56±10^#▴^
normal group	172±21	21±7.5	8.2±5.0	0.0±0.0	70±13

^*^P<0.05, compare with normal group;^#^*P*<0.05, compare with asthmatic group;^▴^*P*>0.05, compare with normal group.

### NK-1R protein expression in the lung

**Figure 2:**
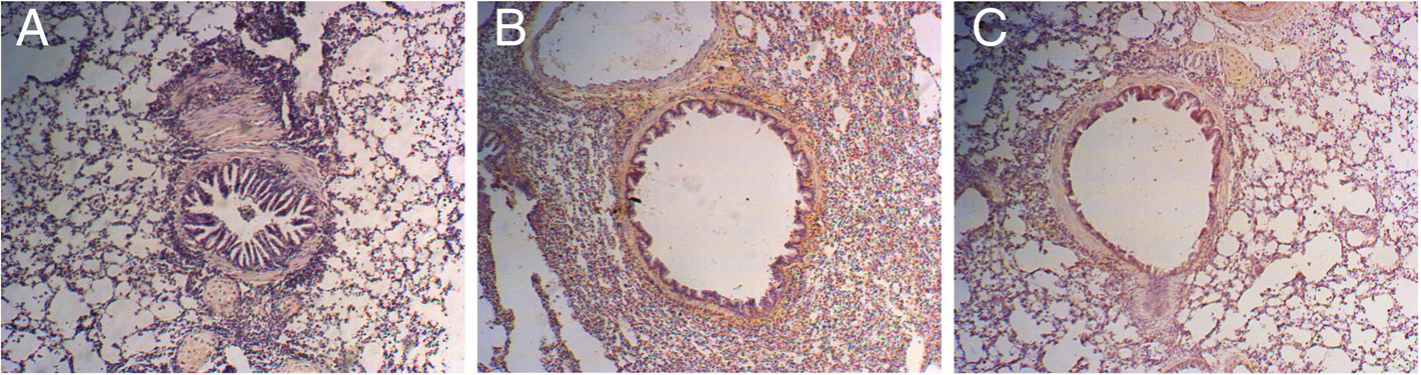
**Immunohistochemical analysis of NK-1R protein expression in the lung from different groups. A**: Representative control group; **B**: Representative asthmatic group; **C**: Representative budesonide treatment group.

Immunohistochemistry was employed to investigate NK-1R protein expression in airways. According to image analysis, the expression of NK-1R protein in the asthmatic group was significantly higher than that in the control group (*P* < 0.05) but this increase was significantly abrogated by budesonide treatment (*P* < 0.05; Figure 2 and Table 2). However, NK-1R protein expression in the budesonide treatment group remained significantly higher than that of controls (*P* < 0.05). 

**Table 2:** NK-1R protein expression in the lung from the different groups

**Group**	**Mean ± SE**	**N**
normal control group	1.0347±0.2503^#^	15
asthmatic group	1.1687±0.1356^*^	15
budesonide treatment group	1.0820±0.1146^*#^	15

^*^P < 0.05, compared with normal group; ^#^*P* < 0.05, compared with asthmatic group.

### NK-1R protein expression in ASMCs

**Figure 3:**
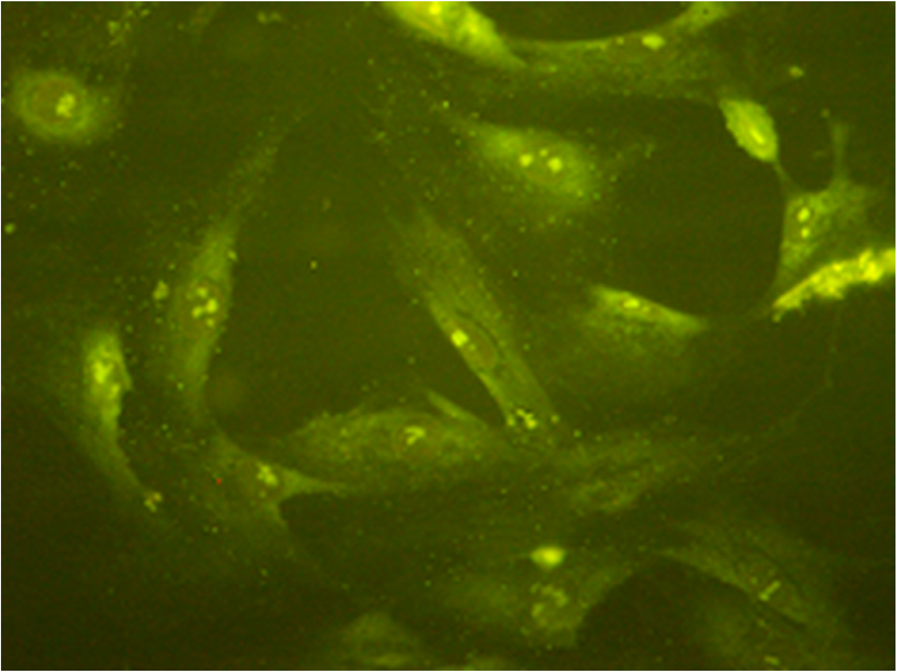
The immunofluorescence of α-actin suggests that the green-stained cells are ASMCs.

**Figure 4:**
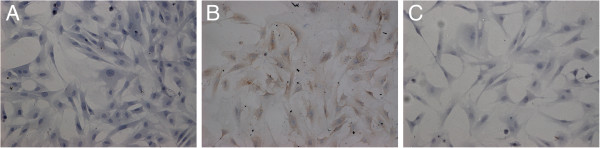
**Immunocytochemical analysis of NK-1R protein expression in ASMCs from different groups. A**: Representative control group cell; **B**: Representative asthmatic group cell; **C**: Representative budesonide treatment group cell. The expression of NK-1R in the asthmatic group (Figure 4B) was significantly higher than that in the control group (*P* < 0.05; Figure 4A) but significantly lower than that in the budesonide treatment group (*P* < 0.05; Figure 4C).

To investigate NK-1R protein expression in ASMCs, we first studied the purity of ASMCs of the fourth passage via immunocytochemistry. Accordingly, the purity of ASMCs was confirmed to exceed 95% by α-actin staining (Figure 3). Image analysis revealed that, as in the aforementioned IHC results, the protein expression of NK-1R in the asthmatic group was significantly higher than that in the control group (*P* < 0.05), but again this level was significantly decreased by budesonide treatment (*P* < 0.05; Figure 4 and Table 3). However, NK-1R protein expression in the budesonide treatment group remained significantly higher than that in the control group (*P* < 0.05). 

**Table 3:** NK-1R protein expression in ASMCs from the different groups

**Group**	**Mean ± SE**	**N**
normal control group	0.3214±0.0181^#^	15
asthmatic group	0.3643±0.0221^*^	15
budesonide treatment group	0.3465±0.0156^*#^	15

^*^P < 0.05, compared with normal group; ^#^*P* < 0.05, compared with asthmatic group.

### NK-1R mRNA expression in ASMCs

Similar to the results observed in all protein expression studies, real-time RT-PCR demonstrated that NK-1R mRNA expression in the asthmatic group was significantly greater than that of the control group (*P* < 0.05) but was significantly lowered by budesonide treatment (*P* < 0.05; Table 4). Yet, NK-1R mRNA expression in the budesonide treatment group was still significantly greater than that of the controls (*P* < 0.05). 

**Table 4:** NK-1RmRNA expression in different group ASMC

**Group**	**Mean ± SE**	**N**
normal control group	1.0347 ± 0.2503^#^	15
asthmatic group	1.1687 ± 0.1356^*^	15
budesonide treatment group	1.0820 ± 0.1146^*#^	15

^*^P < 0.05, compare with normal group;^#^*P* < 0.05, compare with asthmatic group.

## Discussion

In this study, we utilized immunohistochemistry, immunocytochemistry, image analysis, and real-time RT-PCR to analyze NK-1R expression in the airways of asthmatic rats. Immunohistochemistry detected that NK-1R mRNA localizes near the tracheal epithelium superior layer cell surface, mucosa, blood vessel epithelial cells, inflammatory cell surface, smooth muscle cells, and gland cell surface. Furthermore, immunocytochemistry revealed NK-1R expression in the cytoplasm of ASMCs, important cells in the asthmatic airway. Substance P binds preferentially to the NK-1R and induces bronchoconstriction, increases mucus secretion, and facilitates cholinergic neurotransmission, vasodilatation, and plasma leakage [10]. The NK-1R is a member of the tachykinin G-protein-coupled receptor super family, known for influencing a broad array of biological actions, including contraction, secretion, immune responses, and neurotransmission [11].

Glucocorticoids (GCs) are commonly used therapeutic drugs in the treatment of asthma, and they are generally believed to act through an anti-inflammatory effect that involves decreasing inflammatory factors by inhibiting cytokine production and release. Accordingly, budesonide is an inhaled GC that can inhibit inflammation of the asthmatic airway, but the effect of this drug on neuropeptide-induced inflammation in asthma is unknown.

Therefore, in our study, we compared NK-1R expression in the lung and ASMCs obtained from asthmatic rats to those obtained from asthmatic rats treated with budesonide. We found that NK-1R was downregulated in ASMCs, which suggests that budesonide inhibited airway inflammation, at least in part, by downregulating NK-1R expression in ASMCs.

It has been shown that GCs act through both genomic and non-genomic mechanisms. The genomic effects of GCs are generally mediated by cytosolic receptors that alter the expression of specific genes [12]. However, in recent decades, it has been demonstrated that GCs also act through a membrane-initiated, non-genomic mechanism [13-15] that has a rapid onset time, typically within minutes or even seconds after stimulation, in contrast to the genomic action that has an onset time of hours [14]. Furthermore, the genomic mechanism may be mediated through classical GC receptors but may also have a transcription/translation-independent component; these effects might be mediated through some membrane-bound GC receptor that has yet to be identified [13,14], as the action of GCs involves the activation of multiple intracellular signal transduction pathways [16]. Regardless, the rapid non-genomic action of GCs has been extensively reported in the CNS [14], and this action is biologically relevant, as Zhou *et al* previously demonstrated the rapid non-genomic effects of GCs on allergic asthmatic reactions in the guinea pig [17]. Though the mechanism is yet unknown, in the present study, we show that GCs may inhibit airway inflammation through reducing NK receptor expression. However, how and to what extent GCs affect the function of neuropeptides remains to be investigated.

## Conclusions

NK-1R is involved in the pathogenesis of asthma and that budesonide may downregulate the expression of NK-1R in the ASMCs and airways of asthmatic rats, which may alleviate neurogenic airway inflammation.

## Competing interests

The authors declare that they have no competing interests.

## Authors’ contributions

ML carried out the ASMC culture, real-time analysis, immunocytochemistry analysis, image analysis, drafted and revised the manuscript. YS participated in the design of the study, analyzed and interoperated the data, and performed the statistical analysis. All authors read and approved the final manuscript.

## Pre-publication history

The pre-publication history for this paper can be accessed here:

http://www.biomedcentral.com/1471-2466/12/79/prepub
